# Splicing at the phase-separated nuclear speckle interface: a model

**DOI:** 10.1093/nar/gkaa1209

**Published:** 2020-12-18

**Authors:** Susan E Liao, Oded Regev

**Affiliations:** Computer Science Department, Courant Institute of Mathematical Sciences, New York University, New York, NY, USA; Computer Science Department, Courant Institute of Mathematical Sciences, New York University, New York, NY, USA

## Abstract

Phase-separated membraneless bodies play important roles in nucleic acid biology. While current models for the roles of phase separation largely focus on the compartmentalization of constituent proteins, we reason that other properties of phase separation may play functional roles. Specifically, we propose that interfaces of phase-separated membraneless bodies could have functional roles in spatially organizing biochemical reactions. Here we propose such a model for the nuclear speckle, a membraneless body implicated in RNA splicing. In our model, sequence-dependent RNA positioning along the nuclear speckle interface coordinates RNA splicing. Our model asserts that exons are preferentially sequestered into nuclear speckles through binding by SR proteins, while introns are excluded through binding by nucleoplasmic hnRNP proteins. As a result, splice sites at exon-intron boundaries are preferentially positioned at nuclear speckle interfaces. This positioning exposes splice sites to interface-localized spliceosomes, enabling the subsequent splicing reaction. Our model provides a simple mechanism that seamlessly explains much of the complex logic of splicing. This logic includes experimental results such as the antagonistic duality between splicing factors, the position dependence of splicing sequence motifs, and the collective contribution of many motifs to splicing decisions. Similar functional roles for phase-separated interfaces may exist for other membraneless bodies.

## INTRODUCTION

### Phase-separated membraneless bodies

Eukaryotic cells contain many membraneless bodies with distinct nuclear or cytoplasmic localizations (1). These micron-scale bodies were historically characterized by their distinct morphologies and localizations (2). Many of these membraneless bodies, including nucleoli (3–5), Cajal bodies (6) and nuclear speckles (7), are found in the nucleus. Others, including stress granules (8) and processing bodies (9,10), localize to the cytoplasm. Some membraneless bodies are unique to specific cell types; for example, germ granules (11) are found exclusively in germ cells, while synaptic densities (12) are found in neurons (13).

The composition of these membraneless bodies drives their formation through phase separation. Phase separation also describes and explains their biophysical properties and dynamic behavior (2,13–18). Many membraneless bodies are composed of RNA binding proteins, RNAs, and ribonucleoprotein assemblies (19–21). The protein–protein, protein–RNA and RNA–RNA interactions of these membraneless body components play critical roles in phase separation (22,23). The intrinsically disordered, low-complexity regions of many RNA binding proteins form multivalent weak interactions that segregate these proteins together into a separate phase (24). These RNA binding proteins bind specific RNAs through unique RNA sequence motifs. Membraneless bodies are thus often enriched for specific RNA binding proteins and RNAs (1). Furthermore, RNAs tune the fluidity and fusion dynamics of membraneless bodies (25). The principles of phase separation thus not only explain how these membraneless bodies form, but also how they remain stable over long timescales. While membraneless bodies are visibly distinct from other cellular components, dynamic exchange of constituent molecules occurs between membraneless bodies and the surrounding phase (2,26).

Despite a growing appreciation for the role of phase separation in forming membraneless bodies, the functional roles of phase separation remain elusive. We argue that the chemical interface between distinct phases, an emergent property of phase-separated membraneless bodies, enables spatiotemporal organization of biological processes. Specifically, we propose that phase-separated nuclear speckles spatiotemporally organize RNA splicing decisions through the unique chemical environment at their interface.

### Evidence for nuclear speckle function in RNA splicing

Nuclear speckles were first observed by Santiago Ramón y Cajal by light microscopy (27) and later characterized as interchromatin granules by electron microscopy (28). There are a variable number of nuclear speckles in mammalian nuclei of dynamic and irregular shapes (29). Nuclear speckles exhibit the hallmark properties of phase-separated membraneless bodies, including liquid-like behaviors (26,30–33) and dynamic exchange of constituent RNA binding proteins and RNAs with the surrounding nucleoplasm (2,26,30–36).

**Figure 1.**
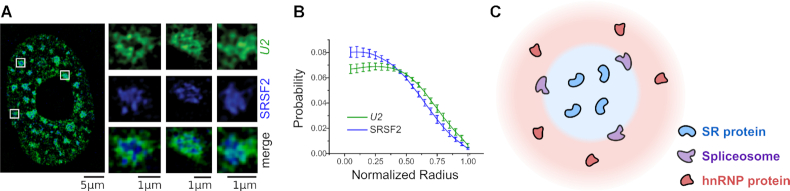
Nuclear speckle composition. (**A**) Structured illumination microscopy (SIM) image of *U2* snRNA (an essential spliceosome component, green), and SRSF2 (speckle-enriched SR protein, blue). Individual nuclear speckles (insets) show localization of *U2* to the periphery of the speckle. (**B**) Probability density distribution of the distance from the speckle center for *U2* and SRSF2. *U2* is distributed further away from the center than SRSF2. (**C**) SR proteins drive nuclear speckle phase separation, shown in blue. hnRNP proteins are excluded from the nuclear speckle and are found in the surrounding nucleoplasm, shown in red. The spliceosomes localize to the nuclear speckle periphery. (A) and (B) adapted from (42) with permission from *The Journal of Cell Science*.

The composition of nuclear speckles was determined by immunostaining and mass spectrometry studies (29,37–42). Nuclear speckles are enriched for SR proteins (29,37,38), a family of RNA binding proteins named for their intrinsically disordered regions of serine and arginine residues (43). Multivalent interactions between low complexity regions of SR proteins play critical roles in phase separating nuclear speckles from the nucleoplasm (1,44). SR proteins are splicing factors, i.e., RNA binding proteins that regulate splicing decisions (45–47). Importantly, another family of splicing factors, the heterogeneous nuclear ribonucleoprotein splicing factors (hnRNPs) (48,49) are excluded from nuclear speckles (39–41). Finally, spliceosomes, the multi-component ribonucleoprotein assemblies that carry out the catalytic splicing reaction, localize to nuclear speckle peripheries (42) (Figure 1A and B). In fact, many speckle-associated spliceosomes contain phosphorylated proteins indicative of active spliceosomes (50). Together, these observations reveal a spatial organization within nuclear speckles: SR proteins are enriched inside the nuclear speckles, hnRNP proteins are enriched in the surrounding nucleoplasm, and spliceosomes localize to the periphery of the speckle (Figure 1C).

In addition to SR splicing factors and active spliceosomes, nuclear speckles are also enriched for RNA. Specifically, the majority of mRNAs transit through nuclear speckles (50–54). Together, these observations suggest that RNA splicing occurs at nuclear speckles. However, the exact role nuclear speckles play in splicing remains a major outstanding question.

### Effects of RNA motifs on splicing decisions agree with nuclear speckle spatial protein organization

RNA splicing occurs in a sequence-dependent manner following logic encoded in the ‘splicing code’ (55,56). The splicing code consists of core sequences required for splicing, including splice site motifs, polypyrimidine tracts, and branch point sequences. Splicing cleavage and ligation occur at splice site motifs, conserved sequences that delineate exon-intron boundaries. Those marking the beginning of an intron are known as 5′ splice site motifs and those marking the end are known as 3′ splice site motifs. In addition, intronic branch point sequences and polypyrimidine tracts are required for intron removal.

While the core splicing sequences are necessary for splicing to occur, they alone do not capture the full complexity of splicing decisions (55,57). In many cases, additional *cis*-regulatory logic determines which splice site motifs are used. This additional logic is encoded in splicing regulatory elements (SREs). SREs are short RNA sequence motifs that are bound by *trans*-acting splicing factor proteins (58–60). In this survey, we focus on the logic encoded in SREs and show how this logic emerges from nuclear speckle spatial organization.

The SREs bound by the two main splicing factor families are enriched in separate gene regions: SR sequence motifs are enriched in exons (61) while hnRNP sequence motifs are enriched in introns (62). This duality in exon and intron motif enrichment also extends to the motifs’ effects on splicing decisions. These effects are typically antagonistic: when one enhances splicing, the other silences (63,64).

Interestingly, splicing decisions are based on the combined contribution of many SREs. In fact, most nucleotides in an exon can contribute significantly to the outcome of splicing (65–67); this presents a conundrum since more proteins appear to be able to bind and affect splicing than could possibly bind concurrently (68).

As will be described below, these and other experimental observations on splicing decisions paint a detailed picture of the complexities of the splicing code. Specifically, they highlight the interplay between SR and hnRNP motifs (55). Yet until now, no molecular mechanism has been proposed that explains these observations.

## THE INTERFACIAL SPLICING MODEL

**Figure 2.**
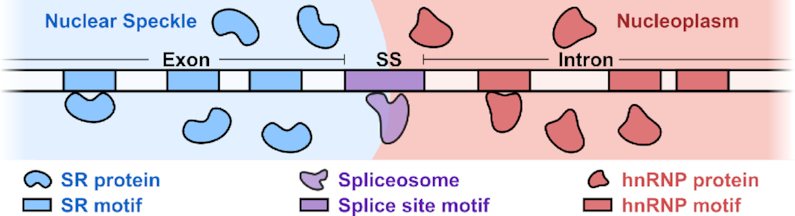
Interfacial splicing model. The SR motif-rich exon is positioned inside the nuclear speckle, whereas the hnRNP motif-rich intron is held outside in the nucleoplasm. The 3′ or 5′ splice site motif (SS) at an exon-intron boundary is positioned at the interface.

We present a mechanistic model that explains the execution of SRE regulatory logic through intramolecular RNA localization at the nuclear speckle interface. Our model hinges on two basic facts. First, there are differences between the chemical environments inside and outside the phase-separated nuclear speckle: SR proteins are enriched inside nuclear speckles whereas hnRNP proteins are excluded to the outside of the nuclear speckle. Second, there are corresponding differences in RNA sequence compositions on either side of the splice site motif: on one side, exonic sequences are SR motif-enriched; on the other side, intronic sequences are hnRNP motif-enriched. Combining these two facts, we arrive at the logical conclusion that an RNA molecule containing a splice site motif flanked by opposing sequence compositions (exonic on one side and intronic on the other) would be positioned so that its splice site motif straddles the speckle interface (Figure 2). Informally, exonic sequences are ‘pulled’ into the speckle, whereas intronic sequences are ‘pulled’ into the nucleoplasm, placing the splice site motif at the interface; this process is not unlike the one driving the positioning of amphiphilic molecules at an oil–water interface. Once the splice site motif is at the interface, it is accessible to the spliceosome, which is also localized to the interface. This, in turn, enables the subsequent splicing reaction.

Our model highlights the functional role of nuclear speckle interfaces in RNA splicing. Importantly, as we will show below, despite its simplicity, the model provides a unified mechanism that can seamlessly explain decades of perplexing experimental observations (Table 1).

**Table 1.** Summary of experimental evidence in support of the interfacial splicing model

Splicing property	Experimental evidence
**Splicing occurs at the nuclear speckle periphery**. Active spliceosomes localize to the periphery. Introns are positioned outside the speckle whereas exons migrate inside.	Immunofluorescence and fluorescence in situ hybridization (69,90)
**Transcripts associate with nuclear speckles in a sequence-dependent manner**. Sequences sufficient for driving transcripts into the nuclear speckle were identified, including intronless SR-enriched transcripts.	Fluorescence in situ hybridization (52,70–73)
**SR and hnRNP proteins have antagonistic effects on splicing decision**. An SR protein bound to a given RNA position typically exerts the opposite effect to an hnRNP protein bound at the same position.	Genome-wide analyses (75,119) and analysis of engineered splicing factors (120)
**Position-dependent effects of SREs on splicing decisions**. SREs have opposite effects depending on whether they are within an exon or an intron.	Mutagenesis experiments (55,121,122)
**Common logic of SREs in diverse splicing decisions**. The same SRE logic governs splicing at 3′ and 5′ splice sites, despite separate spliceosomal components for each.	Massively parallel assays (83) and directed studies of SREs (84,85)
**Collective contribution of many SREs**. Multiple SREs in a broad window around the splice site motif combine to form splicing decisions.	Massively parallel assays (66,67,83,86,87)
**Quantitative biophysical framework explains splicing decisions**. Splicing decisions are based on the total free energy contributions of many SREs and follow the Boltzmann distribution.	Massively parallel assays (83,88)

## EXPERIMENTAL EVIDENCE FOR THE INTERFACIAL SPLICING MODEL

**Figure 3.**
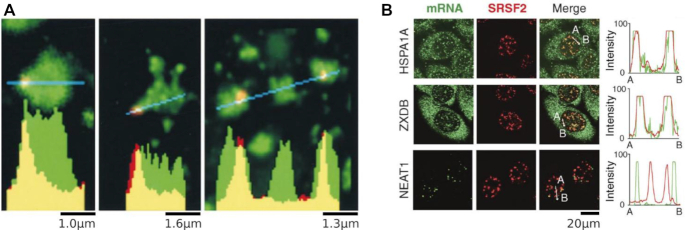
Localization of RNA to nuclear speckles. (**A**) Analysis intron RNA distributions. The collective *COL1A1* intron signal is concentrated at nuclear speckle peripheries. Relatively little intron RNA (red) is detected in the speckle interior (SRSF2, green). Graphs show the fluorescence intensity for pixels along the lines shown in blue. Adapted from (50) with permission from The Journal of Cell Biology. (**B**) Distributions of intronless RNAs at nuclear speckles. Despite not containing any introns, SR motif-enriched HSPA1A and ZXDB transcripts localize to speckles. The long noncoding RNA NEAT1 does not localize to speckles. Graphs show the fluorescence intensity for mRNA and SRSF2 pixels along the lines from A to B. Adapted from (52) with permission from The Journal of Cell Biology.

Two lines of evidence support the idea that the splicing reaction occurs at nuclear speckle interfaces. First, the localization of spliceosomes to the speckle periphery supports this idea (Figure 1). Second, inspection of RNA transcripts that pass through the nuclear speckle reveals a striking feature of intramolecular transcript localization: while introns remain at the speckle periphery (Figure 3A), spliced RNA products migrate into the speckle interior (50,69). This again suggests that splicing takes place at the interface.

In addition, transcripts associate with nuclear speckles in a sequence-dependent manner. Indeed, biochemical studies identified sequences sufficient for driving transcripts to the nuclear speckle (70–73). Notably, nuclear speckle localization occurs independently of the splicing reaction, as transcripts enriched for SR motifs but lacking splice sites still localize to nuclear speckles (52) (Figure 3B). This agrees with our model, in which sequence-dependent intramolecular positioning precedes and facilitates the splicing reaction.

### Sequence-based evidence: duality of SR and hnRNP proteins

Further evidence that supports our model comes from experiments probing how SREs combine to form splicing decisions. In these experiments, splicing products resulting from transfected reporter constructs are quantified. These constructs might contain a fixed splice site motif with various upstream or downstream SREs. This experimental design allows the quantification of the effects of SREs on the fixed splice site usage.

One of the earliest observations from these experiments is the antagonistic effect of SR and hnRNP proteins on splicing decisions. An SR protein bound to a given position in the RNA would typically exert the opposite effect to an hnRNP protein bound at the same position (74).

**Figure 4.**
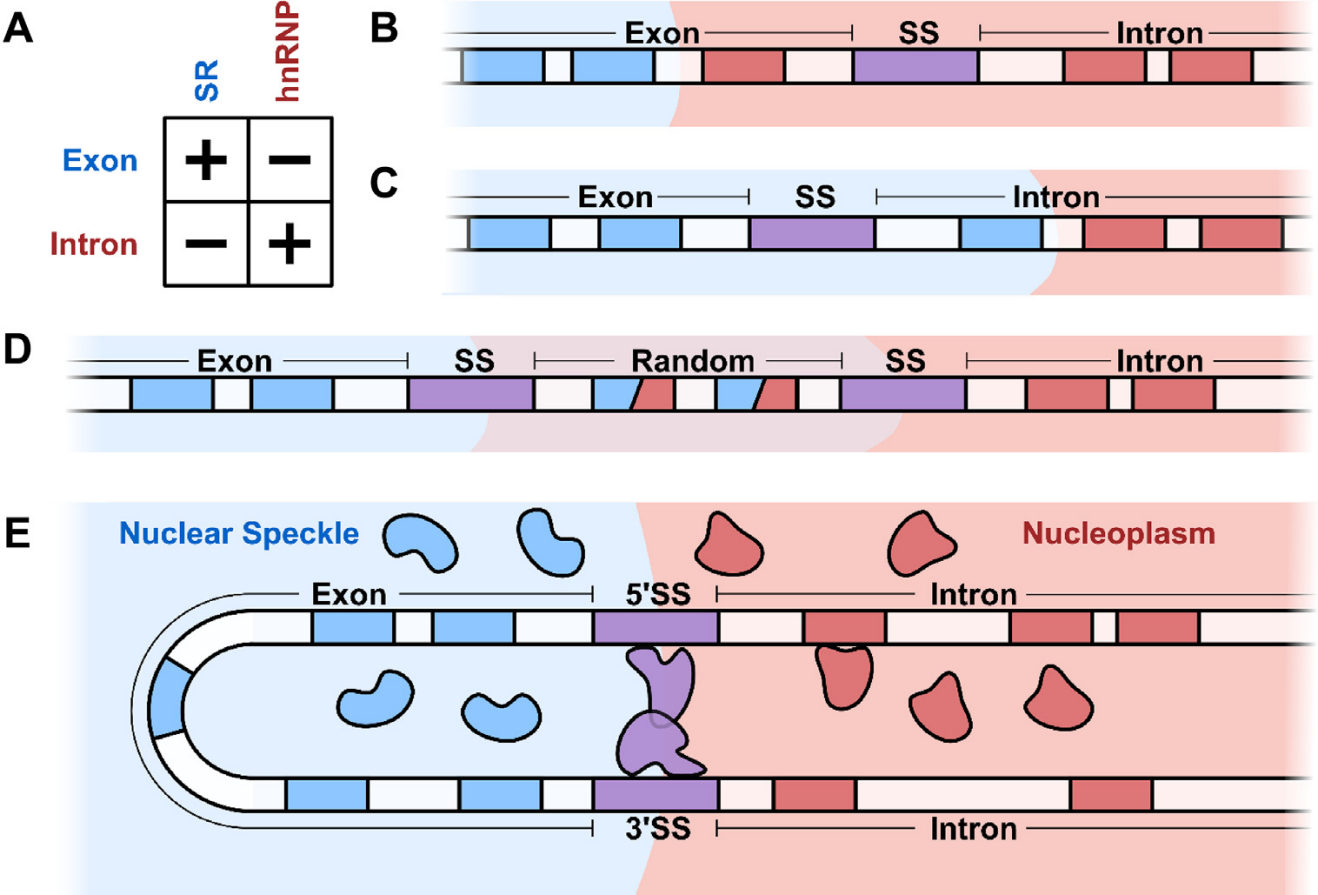
Effects of SRE combinations on splicing decisions. The logic described here applies to both 5′ and 3′ splice sites. (**A**) The effect of SREs on nearby splice sites. SR motifs on the exonic side of splice sites enhance them, whereas those on the intronic side repress them. hnRNP motifs follow a reverse pattern: such motifs enhance splice sites on the intronic side but repress them on the exonic side. (**B**) A schematic depicting how an hnRNP motif on the exonic side of a splice site represses splicing. With the interface upstream of the splice site, the splice site is located inside the nucleoplasm and is less accessible to the spliceosome. (**C**) A schematic depicting how an SR motif on the intronic side represses splicing. With the interface downstream of the splice site, the splice site is located inside the nuclear speckle and is less accessible to the spliceosome. (**D**) The alternative splice site reporter assay. Two splice site motifs are separated by a random sequence containing a mix of SR and hnRNP motifs. The assay includes ∼3$ \times$10^5^ such reporters, each with its own random sequence. Which of the two splice sites is used depends on the balance of SR and hnRNP motifs in the random sequence. If the total contribution of SR motifs is greater than that of hnRNP motifs, then the random region will favor the nuclear speckle phase. This leads to the downstream splice site being positioned at the interface and for it to be used for splicing. Conversely, if the total contribution of hnRNP motifs is greater than that of SR motifs, the random region will favor the nucleoplasm phase. This leads to the upstream splice site being positioned at the interface and for it to be used for splicing. (**E**) Phase separation enables exon definition. By colocalizing both splice sites to the same interface, nuclear speckles help facilitate productive spliceosome assembly.

Moreover, both splicing factor families show a striking position-dependent effect on splicing decisions (Figure 4A) (75). SR motifs on the exonic side of a splice site motif tend to enhance splicing at that splice site (76), whereas such motifs repress splicing if positioned on the intronic side of the splice site motif (77,78). As a consequence, SR motifs are often classified in the literature as exonic splicing enhancers and intronic splicing silencers (55). In contrast, hnRNP motifs show the exact reverse behavior to SR motifs: they repress splicing on the exonic side (79,80) and enhance splicing on the intronic side (81,82). Thus, hnRNP motifs are often termed exonic splicing silencers and intronic splicing enhancers (55).

The fact that both SR and hnRNP motifs exhibit such a position-dependent behavior led to the hypothesis that both families exploit a similar mechanism to determine splice site usage (75). Our model provides such a mechanism. Specifically, an hnRNP motif on the exonic side favors positioning of the splice site outside of the nuclear speckle, making it less accessible to interfacial spliceosomes (Figure 4B). Similarly, an SR motif on the intronic side favors positioning of the splice site within the nuclear speckle, also making it inaccessible to interfacial spliceosomes (Figure 4C).

Importantly, similar SRE logic applies to both 3′ and 5′ splice sites (83–85). This led to the hypothesis that there is a common mechanism shared across SRE-mediated splicing decisions (83). Our model is also consistent with this hypothesis, showing how SRE-dependent nuclear speckle positioning applies to both 3′ and 5′ splice sites.

### Sequence-based evidence: combined effect of multiple SREs

Abundant experimental evidence has demonstrated that splicing decisions depend on a combination of many SREs (66,67,83,86,87). In fact, almost all nucleotides in an exon can contribute significantly to the outcome of splicing (65–67). For instance, one study reported that single nucleotide mutations at >90% of positions in an exon alter splicing (66).

Even though the decision of whether a splice site should be used or not depends on the combination of many SREs, the number of proteins that can possibly bind concurrently to that region of RNA is much smaller (68). Furthermore, it was shown that the protein–RNA interactions between splicing factors and SREs are weak and transient (46). These observations are incompatible with a mechanism in which SREs directly recruit the spliceosome. Instead, another mechanism must combine the information from multiple SREs into a single splicing decision.

Our model provides precisely such a mechanism: it describes how physical space can serve as a medium for combining information from multiple weak interactions into one coherent decision.

### Sequence-based evidence: quantitative predictions

Recent massively parallel reporter assays have enabled detailed quantitative insights into sequence-dependent splicing decisions (83,88). One such reporter assay examined the effects of RNA sequences on splice site choice (Figure 4D). The authors fixed two competing 5′ splice site motifs separated by a short sequence of random nucleotides, allowing the characterization of the effects of each sequence on splice site usage (83). The assay contains ∼3 $ \times$ 10^5^ reporters, each with its own random sequence. The splicing outcome of each reporter is measured multiple times to determine splice site usage statistics. Each reporter is then associated with a ‘splice site usage ratio’, corresponding to the ratio between the two splice site usage probabilities. For instance, if a reporter is spliced with probability 75% at the upstream splice site, and 25% at the downstream splice site, its splice site usage ratio is 75/25 = 3.0. A reporter spliced with probability 20% at the upstream splice site and 80% at the downstream one has a splice site usage ratio of 20/80 = 0.25.

The results of this assay showed a striking pattern. The measured splice site usage ratios closely follow a simple multiplicative law obtained from the independent contributions of individual SREs. Specifically, the authors calculated an effect size score for every possible 6-mer sequence (total 4^6^ = 4096) based on its enrichment in the upstream-spliced reporters. Then, the multiplicative law predicts that the splice site usage ratio of a reporter is given by the product of the scores of all the 6-mers inside the random region. These predictions were found to be remarkably accurate. Moreover, inspecting the scores computed in this analysis reveals a strong agreement with known SREs. 6-mers corresponding to hnRNP motifs have high scores (enhancing splicing in the upstream splice site) whereas those corresponding to SR motifs have low scores (enhancing splicing in the downstream splice site). Similar results were found in an analogous 3′ splice site assay (83); moreover, such a multiplicative law was found to play a universal role in splicing decisions (88).

The multiplicative law is implied by our interfacial splicing model. This can be seen using basic thermodynamic considerations. Indeed, consider a system with two possible states corresponding to the two positions of the splice sites with respect to the interface (Figure 4D). In one state, the upstream splice site is at the interface and the random region is in the nucleoplasm. In the other state, the downstream splice site is at the interface and the random region is in the nuclear speckle. Notice that the regions upstream and downstream of the random region are in the same chemical environment in both states; it is only the random region that changes environments. The free energy difference ($\Delta G$) between these two states can therefore be approximated by the difference in the free energy of the random RNA region between the two environments. This difference, in turn, is given by the sum of contributions coming from the multiple weak interactions between the RNA region and the surrounding splicing factors. Crucially, since splicing factors typically bind short stretches of RNA and do so transiently, we can approximate $\Delta G$ by the sum of $\Delta {G_i}$ where $i$ ranges over all k-mers present in the random region. Accordingly, the splice site usage ratio is given by the Boltzmann distribution as

$$\begin{equation*}{e^{ - {\rm{\Delta }}G/kT}} \approx {e^{ - \mathop \sum \limits_i {\rm{\Delta }}{G_i}/kT}} = \mathop \prod \limits_i {e^{ - {\rm{\Delta }}{G_i}/kT}}.\end{equation*}$$

This mathematical statement agrees with the observed multiplicative law.

## DISCUSSION

### Summary of the interfacial splicing model

While it was previously appreciated that phase-separated membraneless bodies separate and compartmentalize their constituent proteins and RNAs (89), far less attention has been focused on how phase separation also introduces a unique chemical environment at the interface. We proposed that in the case of the nuclear speckle, the interface serves a functional role in executing RNA splicing logic. Our model provides a simple phase-separation mechanism that underlies the regulatory logic encoded in SREs. It describes a rational, functional agreement between *cis*-acting RNA sequences and *trans*-acting splicing factors. Its predictions agree with a large body of experimental results, including the antagonistic duality between splicing factors, the position dependence of splicing sequence motifs, and the collective contribution of many motifs to splicing decisions.

### Phase separation adds logical complexity to RNA splicing

Our model describes how nuclear speckle phase separation confers an ‘order of operations’ (42) to the execution of splicing code logic. First, the RNA is positioned along the nuclear speckle interface based on SRE-encoded logic. Then, the spliceosome carries out the catalytic splicing reaction at the interface based on the logic encoded in the core splicing sequences (such as splice site motifs). This spatiotemporal organization is consistent with a previous suggestion that the execution of SRE logic precedes spliceosome assembly (83).

Several lines of evidence suggest that nuclear speckles are not always involved in the execution of splicing logic. First, nuclear speckles are thought to be sites of post-transcriptional splicing; however, it is clear that splicing also occurs co-transcriptionally outside nuclear speckles at nascent transcripts (90). Second, nuclear speckles are not observed in some organisms despite active splicing (91). In both cases, it is possible that spliceosomes process the RNA substrate directly, without requiring nuclear speckle intramolecular RNA positioning. As a result, we expect splicing decisions in such cases not to follow the complex logic encoded in SREs. Instead, such splicing decisions might be based only on the logic encoded in the core splicing sequences and features such as the organization of genes in chromosomes (92–94) or epigenetic chromatin marks (95–97). To summarize, our model describes how phase separation adds a layer of logical complexity to splicing occurring in nuclear speckles.

### Phase separation enables exon definition

We presented the key tenets of our interfacial splicing model by focusing on the positioning of a single splice site motif to the nuclear speckle interface. However, spliceosome assembly often requires pairing of both 3′ and 5′ splice sites across an exon. This process, termed exon definition (98–100), is distinct from the catalytic splicing reaction.

We propose that our model facilitates exon definition. Specifically, we expect exons, which are enriched in SR motifs, to be fully immersed within a speckle. In this configuration, exons form a ‘U’ shape, with their 3′ and 5′ splice site motifs localized to the nuclear speckle interface (Figure 4E). Colocalizing the two splice sites to the interface reduces the search space from three dimensions to two dimensions, thereby increasing the likelihood of a productive exon-definition interaction between them.

### Open questions related to the phase-separated interfacial splicing model

Our interfacial splicing model raises several questions regarding how unspliced transcripts localize to speckles, how spliced transcripts are released from the speckle, and whether speckles have additional functions that may have crosstalk with interfacial splicing.

First, how are transcripts localized to nuclear speckles? One possibility is that speckles nucleate on transcripts. Another possibility is the active movement of transcripts towards pre-existing nuclear speckles (101,102). It would be of interest to explore both possibilities and whether they relate to our model.

A second remaining open question is how spliced transcripts are released from the nuclear speckle. It is possible that spliced transcripts interact with additional proteins to facilitate their release. Interestingly, the TREX complex, which facilitates transcript nuclear export, localizes to nuclear speckles (103). Moreover, it was previously proposed that transcripts gain export competence by transiting through the nuclear speckle (52). Based on these observations, it appears that association with the TREX complex could facilitate spliced transcript release.

Finally, we remark that in addition to their involvement in splicing, nuclear speckles have been proposed to serve other functions. This includes serving as splicing factor storage repositories (26), transcriptional boosters (101,102,104), chromatin organizers (105,106), RNA quality control centers (52,107), or as hubs for RNA processing (108). Our model does not rule out these additional roles. Further experiments are required to determine if there is crosstalk between interfacial splicing and these other functions.

### Implications for other phase-separated membraneless bodies

**Figure 5.**
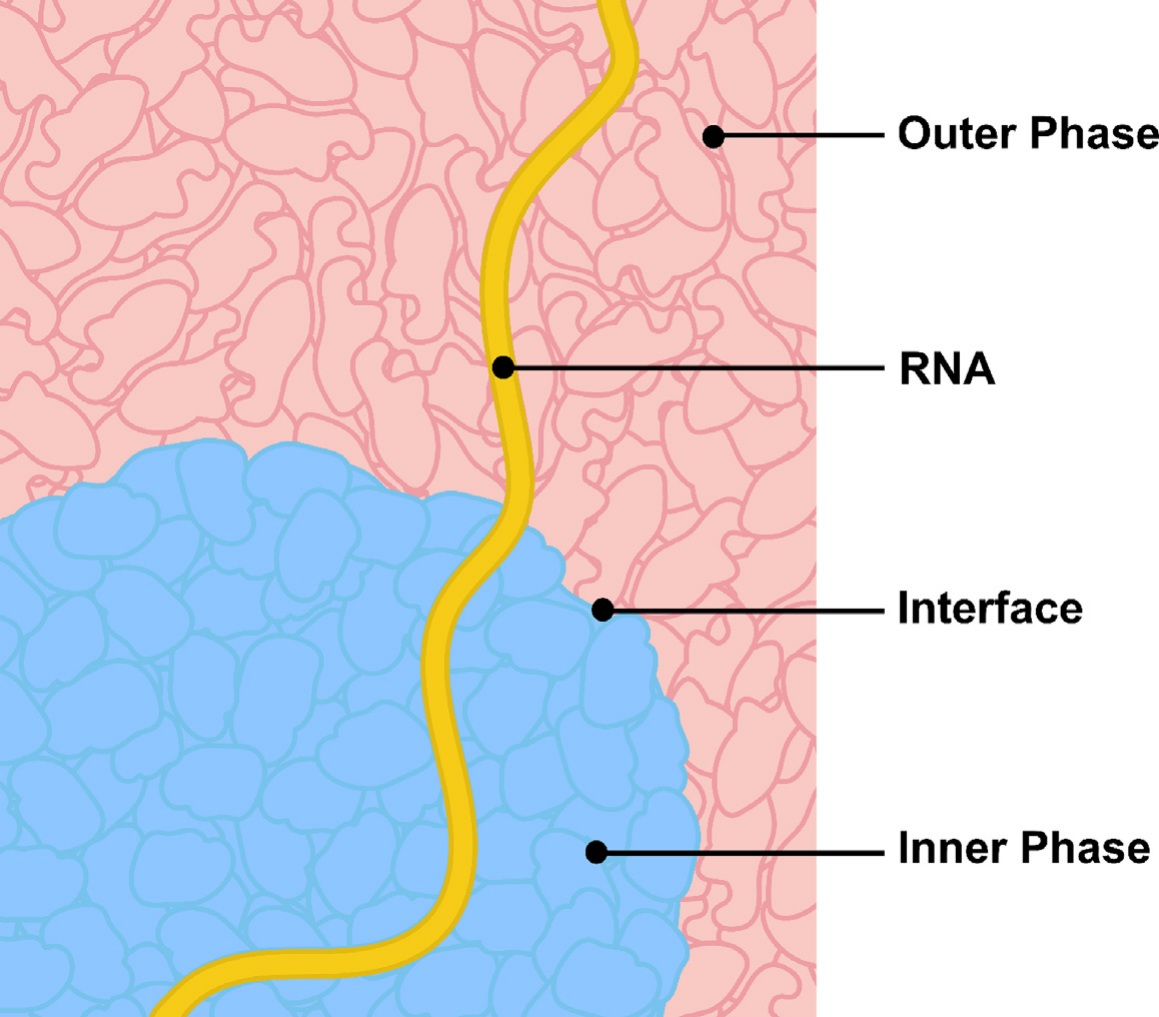
Interfaces of other membraneless bodies might have functional roles in RNA processing.

Based on biophysical first principles, phase separation always results in an interface between the two phases (Figure 5). This should apply to all phase-separated membraneless bodies, not just to the nuclear speckle. Moreover, as is the case for nuclear speckles, studies have shown that peripheries of membraneless bodies, including P granules (109) and stress granules (110), are enriched for specific proteins and nucleic acids. In some cases, peripheries serve as sites for biochemical reactions, such as how Pol I transcription of rRNA in the nucleolus occurs at the border of the fibrillar centers (FCs) and the dense fibrillar components (DFCs) (111–114). It is possible that these peripheral localizations hint at possible functional roles of the interface.

An important question, therefore, is whether interfaces of other membraneless bodies have functional roles. However, experimentally studying the interfaces of membraneless bodies is not trivial (115). To date, the vast majority of studies on phase separation focus on characterizing whether individual proteins exhibit phase separation properties in vitro and in vivo (116). Most of the chemical approaches used in these studies disrupt phase separation and abolish its interface. There is also exciting headway on methods that do not disrupt phase separation, potentially enabling the study of interfaces. For instance, optogenetic methods that activate formation and control size of synthetic phase-separated condensates hold promise for probing the composition and dynamics of phase separation interfaces (117). In addition, high-resolution and quantitative microscopy approaches enable the capture of snapshots of both RNA and protein at phase separation interfaces (42). Similarly, FRAP (110) and inverse FRAP (36) experiments can detect localization to phase-separated interfaces.

Here, we also make the case that sequence logic can identify signatures of localization to a phase-separated interface. Therefore, analyzing sequence logic can complement microscopy-based approaches. A powerful modern approach to understanding such logic is through the use of massively parallel reporter assays (118). These assays were invaluable in informing our model.

As the field advances toward investigating functional roles for phase separation, we anticipate that more focus will shift to emergent properties of phase separation such as phase separation interfaces. These interfaces may hold the key to understanding functional roles of other membraneless bodies, a central question in the field. Inspection of previous work combined with state-of-the-art techniques will be critical to probing this question.
